# Improving Employment Through Interpersonal Psychotherapy: A Case Series of Patients With Treatment-Refractory Depression

**DOI:** 10.3389/fpsyt.2021.617305

**Published:** 2021-04-23

**Authors:** Takeshi Katagiri, Yoshikazu Takaesu, Mariko Kurihara, Yuki Oe, Miho Ishii, Naoko Onoda, Tomonari Hayasaka, Yuta Kanda, Yayoi Imamura, Koichiro Watanabe

**Affiliations:** ^1^Department of Neuropsychiatry, School of Medicine, Kyorin University, Tokyo, Japan; ^2^Department of Occupational Therapy, Faculty of Health Sciences, Kyorin University, Tokyo, Japan

**Keywords:** treatment-refractory depression, interpersonal psychotherapy, employment, major depressive disorder, psychotherapy

## Abstract

Patients with treatment-refractory depression (TRD) have significantly great losses in work productivity and employment. Interpersonal psychotherapy (IPT) is considered an approach for the treatment of TRD. However, the effectiveness of IPT in patients with TRD remains unclear. In this study, we report cases of TRD patients who underwent IPT after a detailed evaluation, along with their employment status. Of 112 patients who experienced 1-week examination administration for TRD at Kyorin University Hospital, which aimed to determine appropriate diagnosis and treatment approaches for each patient, four patients who met the criteria for major depressive disorder according to DSM-IV-TR and were determined suitable for IPT were included in this report. Two patients had moderate, one had mild, and one had remission levels of depressive symptoms according to the Montgomery-Asberg Depression Rating Scale at the time of admission. All four patients completed the scheduled sessions of IPT (6–16 sessions) in the outpatient clinic and achieved remission. All four patients attained full-time employment within 6 months after receiving IPT. This study suggests that the appropriate selection of IPT might be effective for TRD patients, possibly leading to positive outcomes, including work productivity and employment status.

## Introduction

Depression can often lead to remission and recovery with standard therapeutic interventions in patients, but there are cases in which long-term remission is not achieved. For example, the U.S. STAR^*^D study results have demonstrated that approximately one-third of patients are refractory over 48–60 weeks (1). Secondary analyses of the STAR^*^D study also reported that losses in functionality, employment, and productivity were significantly higher in patients with treatment-refractory depression (TRD) (2). Cross-sectional assessments have reported that TRD patients experience impaired work-related activities, impairment of favorable personal relationships, and low involvement in recreational activities (3). A meta-analysis of interpersonal relationships and workplace employment for healthcare professionals (4) indicated that employment was unstable due to interpersonal conflicts, mental distress, and low social support in the workplace. Therefore, it is considered that there are mutual influences between TRD, interpersonal relationships, and employment.

Interpersonal psychotherapy (IPT) was developed by Klerman and Weissman in the US in the 1970s (5). It is an evidence-based short-term psychotherapy that focuses on the “interpersonal relationships” of patients with depression and has demonstrated positive effects on depression (6). Recent studies have shown that IPT is effective for illnesses such as eating disorders, perinatal depression, social anxiety, adolescent depression, bipolar disorder (7), and PTSD (8). Pharmacological therapy is generally the first-line treatment for moderate to severe depression. However, the 2009 National Institute for Health and Care Excellence (NICE) guidelines recommend the introduction of psychotherapy in addition to drug adjustments when sufficient efficacy is not achieved (9). Different types of psychotherapy have been used for TRD and IPT is one of them, but no clear conclusion has been made regarding its effectiveness (10, 11).

The treatment policy for TRD patients in our facility was decided by detailed assessments conducted for each individual case of TRD. Improvements were seen in all four patients who were deemed to have been good indications for IPT among these patients, and they were able to return to work. This study reports on these four patients, including a discussion.

## Methods

### Subjects

The Neuropsychiatry Department of Kyorin University Hospital implemented testing and hospitalization programs to consider TRD diagnosis, deciding upon treatment policies, and conducted research to investigate the actual circumstances of TRD. Patients who had been diagnosed and treated for major depressive disorder but were suffering from a prolonged depressive state and the inability to maintain stable employment for more than 6 months were eligible for the TRD test. This study was approved by the ethics committee of our hospital. From April 2015 to September 2018, a total of 112 patients from all over Japan participated in testing and hospitalization for TRD. Of these, four patients were accessible, and IPT was recommended and requested by the patient. IPT was performed only for these four patients.

The certified psychiatrists' review, Wechsler Adult Intelligence Scale-III (WAIS-III), House-Tree-Person-Person Test (HTPP), imaging examination (head computed tomography, chest and abdominal X-ray), self-administered questionnaire, Mini-International Neuropsychiatric Interview (MINI), and occupational therapy were provided during the week of hospitalization. Patients with one of the four patterns raised in the interpersonal relationship issues of IPT (sorrow, discord over roles in interpersonal relationships, changes in personal role, and deficient interpersonal relationships), such as patients who did not have any close emotional supporters due to discord and those who exhibited depressive symptoms due to changes in their own roles, were selected upon reviewing detailed medical history interviews and life charts. We also excluded patients with episodes of psychotic symptoms, obvious developmental characteristics, or intellectual problems. All findings were organized and comprehensively investigated in a multidisciplinary conference, upon which the indications for IPT were determined. The test findings were summarized in a report, and feedback was provided to patients for ~1 h.

The medication changes at the beginning and end of IPT were as follows: In Case 1, escitalopram 20 mg was tapered off and no oral medication was administered. In Case 2, the dose of lamotrigine was increased from 25 to 100 mg. In Case 3, the dose of lamotrigine was increased from 50 to 150 mg. In Case 4, the dose of venlafaxine hydrochloride 37.5 mg was increased to 75 mg.

### Intervention

**Figure 1:**
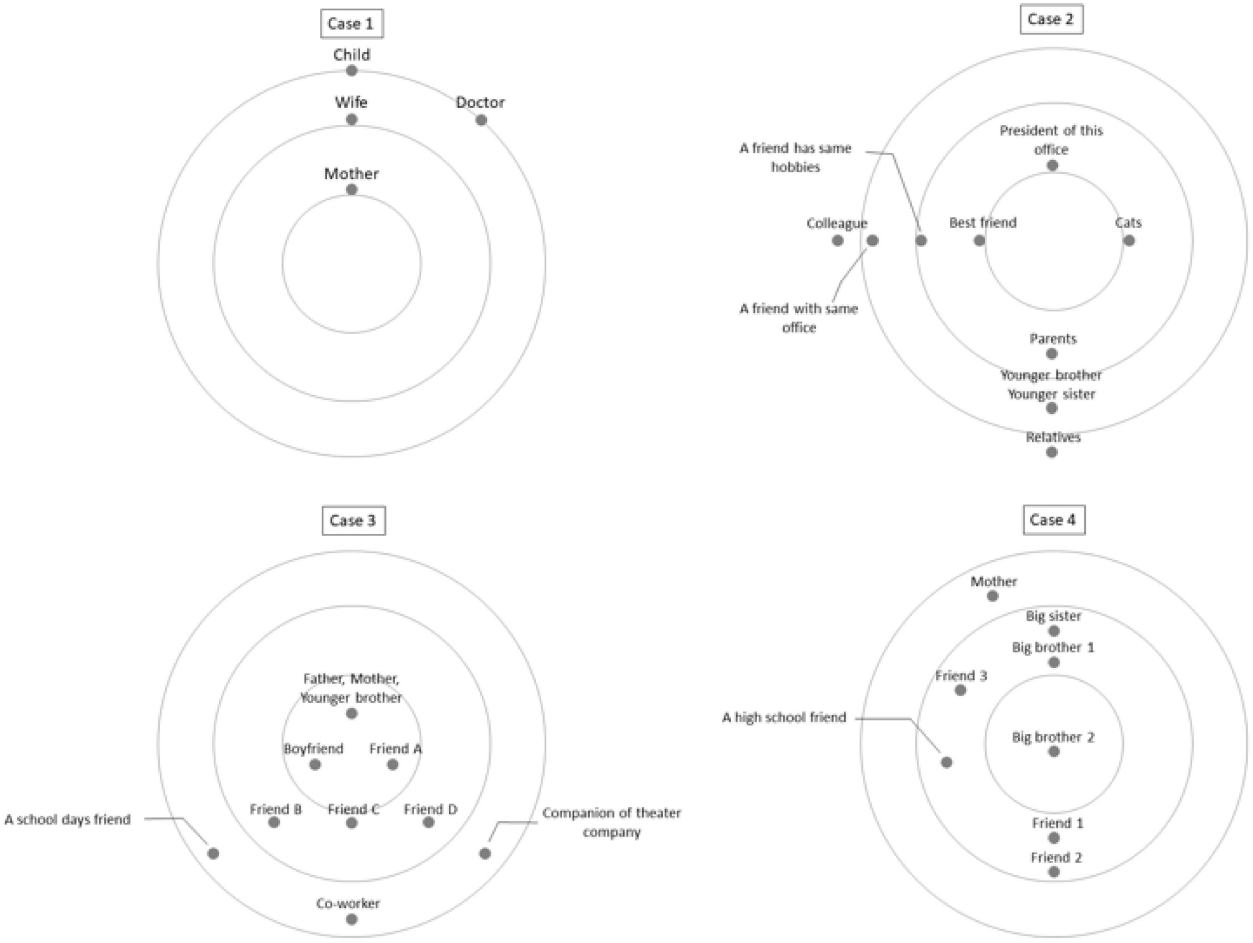
Graphical representation of interpersonal circles. For the interpersonal inventory, we used a simple 3-concentric circle graphic representation of an iterpersonal network. The interpersonal circle of each case, focusing on the self, describing the influential others, and lookiing at the presence and current relationship of significant others.

IPT was conducted by one therapist (first author, TK). He had 13 years of psychiatric clinical experience and 10 years of IPT treatment experience and had received training in IPT mainly through group supervision organized by IPT-JAPAN certified by the International Society of Interpersonal Psychotherapy (ISIPT). IPT was performed according to a manual published by Klerman et al. (5). Interpersonal relationships can be a source of stress. At the same time, the psychological states created by stress can distort interpersonal relationships. Interpersonal psychotherapy focuses on interpersonal relationships, particularly current relationships with “significant other(s)” who have a significant influence on emotional well-being. It aims to understand the relationship between symptoms and interpersonal relationships, identify methods to address interpersonal issues, and enable patients to practice these themselves. We conducted a detailed interpersonal inventory and categorized four interpersonal problem areas (grief, interpersonal role disputes, role transitions, and interpersonal deficits) that contribute to the onset and maintenance of symptoms. Interventions were implemented according to the strategies recommended for each problem area. As the patients gained communication skills through the sessions, they were able to resolve interpersonal problems and conflicts on their own and obtain the interpersonal support they needed, reducing their depressive symptoms in the process. The interpersonal circles of each patient are illustrated in Figure 1. The interpersonal circle is a diagram in which the self is placed in the center and others are placed in order of closeness to the individual; this is an essential task when considering differences in expectations and interpersonal issues in IPT. One session was 50 min and once a week as a general rule, but individual circumstances, such as working conditions, were taken into consideration for the schedule.

## Results

### Characteristics of Patients Who Underwent IPT

Detailed medical history interviews using growth history, present medical history, life charts, structural department interviews, and various psychological tests were conducted for TRD patients in our hospital. Characteristics of the four patients for whom IPT was subsequently implemented are summarized in Tables 1, 2. All cases were diagnosed with major depressive disorder using the MINI on admission for TRD testing, but all did not meet the criteria for a major depressive episode at the end of the IPT session.

**Table 1:** Medical history of participants.

**Case**	**Sex**	**Age**	**Age of Onset**	**Medical history**	**Life history**	**Progress**	**Medication**	
							**At the start of IPT**	**At the end of IPT**
1	M	48	44	Childhood asthma	Good grades when going to school. Struggled with group activities. After graduating from a science university, he worked for 10 years as a science specialist. He was transferred in 2004 and felt that his work was worthwhile. He is married and is part of a four-member household (wife, two children, self).	Marital relationship deteriorated in 2013 when the wife became busy with her own work and raising children. Depressive symptoms appeared in the same year after the patient was promoted. Patient was diagnosed with depression at a nearby psychiatric clinic in January 2014. But the condition improved in December of the same year and the treatment ended. Patient was found to have relapsed depressive symptoms in April 2015 due to interpersonal conflict in the workplace, and outpatient visits resumed. Depressive state was subsequently prolonged in a perturbed fashion due to interpersonal conflict with wife and at the workplace. TRD examination was performed in July 2017.	ESCI 20 mg SULP 100	No oral administration
2	F	41	39	Asthma Atopic dermatitis	Patient felt the stress of interpersonal issues with student friend since high school. Admitted to medical vocational school in 2013, worked as a medical worker since April 2016. Unmarried, living alone.	Patient was reprimanded by senior due to work issues in January 2017, after which depressive symptoms appeared, and developed a tendency to skip work. Diagnosed with depression in nearby psychiatric clinic in February 2017, took leave from work in March of same year. Depressive symptoms improved in May 2017 and returned to work. However, depressive symptoms relapsed shortly afterwards due to reprimand from senior, and took leave from work in June of same year. TRD examination was performed in September 2018.	ARIP 2 mg LAMO 25 mg ZOLP 5 mg ESTA 1 mg	ARIP 2 mg LAMO 100 mg ZOLP 2.5 mg
3	F	23	20	Allergic rhinitis	Patient was active when she was a student, and she went to junior college in order to study theater. Patient appeared on stage while working part-time in a restaurant after graduation, and periodically auditioned. Lives in a family of four (father, mother, younger brother, self).	Depressive symptoms appeared due to verbal argument with mother in August 2015 over issues at work. Consulted nearby psychiatric clinic in October of the same year and began outpatient visits. Depressive symptoms improved in January 2016 and hospital visits were suspended. Patient began living with partner in March 2017, but depressive symptoms relapsed due to interpersonal conflict with partner. Hospital visits resumed in October 2017, but depressive symptoms persisted. TRD examination was performed in March 2018.	LAMO 50 mg	LAMO 150 mg PERO 12 mg
4	M	23	14	–	Parents had a poor relationship since patient's childhood, and there was a strong aversion toward parents. Family environment deteriorated following father's loss of employment and patient's grades dropped. Patient had few friends during school and poor interpersonal interactions. His final academic history was college graduate, and he was employed at an IT firm following graduation.	Unemployed father began to become violent at home in 2008, when patient was in junior high school, and patient's depressive symptoms began to appear at this time. Patient was employed at IT firm in April 2017. Depressive mood, insomnia, cognitive slowing, and nausea developed. Depressive symptoms were prolonged even when patient consulted nearby psychiatric clinic in July of the same year and began outpatient visits. TRD examination was performed in April 2018.	VENL 37.5 mg SULP 100 mg ESZO 3mg	VENL 75 mg SULP 50 mg

*IPT, Interpersonal Therapy; ESCI, escitalopram; SULP, sulpiride; ARIP, aripiprazole; LAMO, lamotrigine; ZOLP, zolpidem; ESTA, estazolam; VENL, venlafaxine; ESZO, eszopiclone; PERO, perospirone*.

**Table 2:** Summary of each patients examination result and final diagnosis.

**Case**	**MADRS**	**YMRS**	**Maudsley TRD scale**	**MINI**	**WAIS-III IQ**	**AQ**	**ASRS**	**HTPP**		**Final diagnosis**
								**Feature**	**Psychological characteristics**	
1	26	0	6	- Major depressive - Episode - Obsessive-Compulsive - disorder Generalized anxiety disorder	- FIQ: 112 - VC: 109 - PO: 101 WM: 115 - PS: 102	26	1	Cotton candy tree crown/A large number of windows/Uneasy stroke/Sun/Branch of trunk to Line/Anxious person Image/Distorted shoulders/Erase the mouth of a female image	Contrary to his unfriendly attitude, he has strong internal anxiety and lacks self-confidence. In his heart, he wants to get along well with others, and he is trying, but he is clumsy and he cannot get around. Impulsive and self-explanatory tendencies can be seen. There seems to be much anxiety about his wife's aggressiveness.	- Major depressive disorder - GeneralizedAnxiety - disorder
2	4	0	7	- Subtype - Depression	- FIQ: 112 - VC: 109 PO: 119 WM: 85 PS: 92	17	2	Cotton candy tree crown/Simple house/Exposed windows/Apple without fluff/Expansion of hands/Beautification, person image with distance from reality	Avoidance and defense are strong, but in the heart she seeks a gentle relationship. She has a strong vanity that she wants people to see her well. So, in a situation where things go wrong, she may be confused.	Major depressive disorder
3	19	1	7	- Major depressive - Episode	- FIQ: 117 - VC: 122 PO: 119 WM: 88 PS: 121	24	4	Cotton candy tree crown/Streamline Trees/Hands like gloves/Childish and doll-like figure/Spotted eyes	Ego is unstable and easy to be swept around. Although fits in superficially, she is unable to put herself out internally and accumulates anger; it is easy for her to experience stress in interpersonal situations.	Major depressive disorder
4	24	1	6	- Melancholy - Subtype - Depression - Episode	- FIQ: 116 - VC: 120 PO: 106 WM: 119 PS: 113	34	3	Cotton candy tree crown/Unbalanced trunk and crown/Apples/Flowers that snuggle up to the trunk/Emphasis on masculinity/Waist pose on hand/Female image with open arms	The way of taking interpersonal relationships is extreme. It is unstable, such as taking a distance or being too dependent. Since there are many ideals and desires, there seems to be extensive conflict in the real world. It is difficult to adjust masculinity and to control the desire for dependent affection; a good target for identification is required.	Major depressive disorder

*MADRS, The total score of Montgomery Asberg Depression Rating Scale; YMRS, The total score of Young Mania Rating Scale; TRD, Treatment-Refractory Depression; MINI, Mini Mental State Examination; WAIS-III, Wechsler Adult Intelligence Scale-III; AQ, Autism-Spectrum Quotient; ASRS, ADHD Self-Report Scale; VC, Verbal Comprehension; PO, Perceptual Organization; WM, Working Memory; PS, Processing Speed; HTPP, House-Tree-Person Personality Test; IPT, Interpersonal psychotherapy; IPSRT, Interpersonal and Social Rhythm Therapy*.

### Intervention Course

All four patients were willing to participate in IPT when test-based feedback was conducted; IPT was then introduced as a treatment option, and intervention was performed. Patients whose so-called “initial IPT work” was completed (from medical history to interpersonal relationship inventory during TRD testing) had shorter subsequent IPT sessions, and treatment was concluded after a total of 6–16 sessions.

### Case Studies

#### Case 1

The patient was originally aware of his awkward interpersonal relationships, but the cause of his current depressive symptoms was thought to be due to interpersonal stress in the workplace. It was explained to the patient during feedback of testing and hospitalization results that relationships with not only everybody in the workplace but his wife in the same kind of work were explained as a major factor. He seemed surprised, but he was interested in a new perspective that was different from previous approaches, and he requested IPT.

The patient started IPT in September 2017, and the MADRS score was 26. The patient worked several days a week on a part-time basis at the start of IPT, but little work was assigned to him, and he felt inadequate and discouraged. His original social functions were high, but he had a high degree of inner anxiety. He began to experience depressive episodes when he was burdened with work once discord manifested between him and his wife, with whom he had a superficially good relationship. We shared a treatment policy to correct the gap in role expectations with his wife and getting out of gridlock with her to try to stabilize his mood and achieve stable employment. The therapist first explained these policies to his wife, but no cooperation was obtained from her. Therefore, we sought to create conversation time between the patient and wife, which at the time was non-existent. During the process, the wife was dissatisfied that the patient would not discuss difficulties with her during challenging times at work, and that she did not agree with the division of child-rearing work between them. The gridlock gradually disappeared as the patient's comprehension of the wife's characteristics and expectations deepened, and he obtained communication skills. A cooperative relationship between work support and child-rearing was formed as a result of renegotiation between the two individuals. The wife began to cooperate with the patient in starting a new business, and at this stage in February 2019, the 16 IPT sessions were concluded. The patient also succeeded in starting his own business in June of the same year.

#### Case 2

The patient had experienced major changes from her conventional living environment, had major decreases in motivation, and was unsure of what to do. The patient initially blamed others and thought that the cause of her depression was that people around her did not support her. She felt that the treatment strategy of IPT, which resolves present problems that impact mood (e.g., workplace and lifestyle troubles) with a focus on interpersonal relationships, was refreshing and felt motivated for treatment.

The patient began IPT while on leave in October 2018, and the MADRS score was 4. Although the MADRS score was very low, as the course of the disorder was prolonged with recurrence, employment was not stable, and the patient felt strong distress over her decreased social function. We thought that IPT would be useful for the patient because her employment was not stable even if the rating scale did not indicate depression. Interpersonal patterns involving a small number of people and minimal emotional interaction were observed in the patient; the problem lies in the fact that the patient was unable to request support from others. Another issue was that the workplace required closer and more emotional interpersonal relationships. The goal of acquiring communication skills where necessary support can be obtained from surrounding people, even under stressful conditions, was set, and the patient consented.

The patient felt stuck because she was unsure about how to negotiate the issues of organizing multiple lessons, such as ballroom dancing, which became a burden because the patient could not just quit, and of helping at the office where she was commuting as part of rehabilitation. Therefore, adjustments were made while conducting roleplay, and we sought to improve communication skills. The patient also suffered from continuous sleep cycle disturbances because she was making a livelihood from part-time work, and an increased tendency of depressive symptoms was seen for several days after partying with her friends at weekend events and returning home late at night, so social rhythm therapy was also introduced. This process improved her depressive symptoms, and she returned to work in March 2019.

There was a stressful event even after returning to work where a colleague had taken a high-handed attitude, but the patient's emotions and ways of expressing her thoughts were examined during roleplays and implemented. The patient still had struggles facing her strong-minded seniors, but she was able to discuss with her superiors when she felt emotional struggles, and the 16 IPT sessions were concluded in May 2019.

#### Case 3

The patient has ongoing unstable relationships with her mother, partner, and other close individuals, and she perceived difficulties in intimate relationships, but the issues were unclear. The patient was able to deepen her understanding of her weaknesses in interpersonal relationships after obtaining visual information from a drawing test (House-Tree-Person-Person test) conducted during testing and hospitalization feedback. She demonstrated interest in IPT.

The patient began IPT in May 2018, and the MADRS score was 19. She would occasionally be involved in part-time work and had no regular employment. She always had a poor relationship with her mother, did not have strong emotional interactions with other family members, and had attachment issues. Problems in human relationships have caused strong confusion in patients. She lived with her significant other but decided to return home after discord with her partner. The goals of understanding the mismatched expectations with her mother and significant others, and learning appropriate self-expression without being excessively concerned about other people's judgment, and learning communication skills were set, and the patient consented to this goal. She began to sort her mother's personal issues and her own issues while accepting her anger toward her mother, which allowed her to gradually put an appropriate distance between them; this resulted in less emotional instability due to her relationship with her mother. However, the patient's depressive symptoms worsened after a verbal argument with her significant other in October of the same year, and she was admitted to our hospital for 9 days. We set up sessions that also involved her significant other during this hospitalization period, and we sought to repair the mismatched expectations by having the two individuals share what they wanted from the other partner, and establish a number of rules to continue their relationship. Subsequent stabilization of her relationship with her partner resulted in a gradual stabilization of her mood, and she began to show more motivation toward work. She finished her 12 sessions in December of the same year. She was hired as a full-time employee in March 2019.

#### Case 4

The patient had always been aware of his awkwardness in interpersonal relationships, but he had little insight as to how this affected his depressive symptoms. These problems were objectively exhibited during testing and hospitalization feedback, and deepening the understanding of the associations between improved interpersonal skills and depressive symptoms served as a motivation for conducting IPT.

He retired in May 2018, as decided before the introduction of the IPT was considered. The patient began IPT in June 2018, and the MADRS score was 24. It was observed that family functions were inadequate, there was little emotional interaction, and he had only superficial relationships when he was a high school student. The patient wanted interpersonal relationships but had few opportunities to cultivate communication skills, and it was also thought that attachment issues were causing awkward interpersonal relationships. The patient was unable to take the necessary communicative steps in various social settings even after employment and exhibited maladaptive habits. The patient started a new job at another company shortly after starting the sessions. Here, the goal of being able to address personal and professional issues while learning interpersonal skills to continue stable employment was set, and sessions were continued. Other individuals who could provide support, such as his brother and workplace seniors, were recruited, and we sought to develop communication skills in the patient by conducting roleplays in various difficult settings. The patient was able to stabilize emotionally as a result of creating allies in the workplace, and he was able to address issues even when they arose in the workplace. Sufficient improvements in depressive symptoms were also observed when his responses in the workplace improved. The patient was able to obtain sufficient information needed for IPT introduction during testing and hospitalization interviews, so the six IPT sessions were concluded in November of the same year.

## Discussion

In this study, various tests were conducted on TRD patients, indications for IPT were examined based on these findings, and the course of four patients was summarized. Our results revealed that all four patients were able to return to work, and it was shown that IPT was effective when indicated for TRD patients. Each patient had favorable motivations for conducting treatment according to the formulation, and there were no patients who suspended treatment. The intervention period and the number of sessions varied, but all patients returned to work and found stable employment either during IPT treatment or within 6 months of concluding treatment.

Refractory to treatment is subjective and often does not correspond to objective measures. The four patients in this study also participated in testing and hospitalization for TRD to resolve the distress of their dissipating depressive symptoms and unstable working conditions. Based on the results of each test, a multidisciplinary conference was held, and interpersonal problems were considered to be a major factor in the lack of improvement in social functioning. Therefore, IPT, another treatment option, was proposed and agreed upon. Although the outcome of this study is the social function of employment, there is a lack of data on objective measures of depressive symptoms, social adjustment, and quality of life pre-post treatment. The lack of such data is a major limitation of this study. Second, in Cases 2, 3, and 4, it cannot be denied that the effect of increasing the dose of the drug may have contributed to the outcome, which is a limitation of this study. Third, baseline social functioning prior to individual IPT implementation is not uniform and can be considered a confounding factor.

In this study, we considered why IPT results in improvements in TRD and employment status for the four patients. A common factor across all four patients was difficulty in their work and personal lives. IPT revealed the subject individuals who they struggled with, and the issues in their own relationships with significant others were clarified. The patients were able to achieve self-realization. Being able to recognize that the problems they face were caused by relationship issues with themselves and significant others was the trigger for escaping their TRD. Patients were able to receive the necessary support from significant others through this process and move toward improvements in depressive symptoms and stable employment. It is generally difficult for patients to feel resistance against IPT because its formulation focuses on recovering social function while remaining close to the patients' own emotions. The various objective feedback of the test results, as was done in this report, was thought to enable a more specific and objective understanding of the relationship between the patient and interpersonal relationship patterns. This was thought to be a major motivation for treatment. Although the effect of IPT on typical depression has been demonstrated in previous studies, few studies have investigated the effect of IPT with a focus on TRD. Souza randomly assigned 40 patients with TRD between the standard treatment and IPT groups in a randomized controlled trial to compare the degree of improvement in depressive symptoms. Human development and social relations (HDSR), Beck Depression Inventory (BDI), and Clinical Global Impression (CGI) improved at 8, 12, 19, and 24 weeks in both groups, but there were no significant differences between the groups at any time point (10). The 2018 Cochrane Database Systemic Review also tested a variety of psychotherapies for TRD and showed that the addition of psychotherapy, including IPT, to usual care, could help improve short-term symptomatic response and remission rates. Most evidence on medium-term and long-term usefulness comes from a single trial, and no clear conclusions are available (11). Therefore, it is difficult to say that the usefulness of IPT for TRD is still established. The background to this is that there are diverse elements that contribute to the intractability of depression, including problems in interpersonal relationships, and it is thought that uniform improvements with an identical treatment method are difficult. Of the obtained TRD test results, we excluded patients with psychiatric symptoms or intellectual problems from indications of IPT, as well as patients who had little awareness of interpersonal relationship problems (e.g., those with autism spectrum disorders, schizoid personality traits) and those in whom other treatment methods are desirable considering future communication abilities. Psychological tests are useful in understanding the characteristics of such interpersonal relationships. The House-Tree-Person-Person Test (HTPP) is a projection method and is a modified version of the figure painting in the HTP test developed by Buck (12), which is a psychological test that depicts both male and female figures in addition to pictures of a house and tree (13). The analysis of these results extracts the level of awareness of interpersonal relationships and unstable interpersonal relationship patterns (e.g., important people who impact the patient and the emotions associated with them, the method by which those emotions are expressed), based on Takahashi's references. Projection methods such as HTPP have conventionally been used as assessment tools and for evaluative objectives of treatment methods, but positioning them as decision materials for indicating psychiatric treatments as was done in this study is a new initiative. Although all four patients who took up had conflicts with their interpersonal relationships and communication ability, these problems were not linked with their own symptoms or social functioning problems. In a follow-up survey of Japanese workers returning to work after an episode of major depressive disorder 1 year later, 50.5% continued to work, but the reason for their sick leave included relapse, retiring for personal reasons (e.g., interpersonal relationships, work environment stress, and family relations), being laid off or fired (14). Moreover, patients with TRD had worse work productivity and social functioning than non-TRD patients (2). Two years have passed since the end of IPT, and all cases have continued to work, suggesting that the specific approach to interpersonal relationships and social functioning through IPT, which had not been intervened in the past, was effective.

Various testing feedbacks are thought to be useful as a motivator for psychotherapy. It has been reported that highly motivated patients with generalized anxiety disorder were able to benefit significantly more from psychosomatic therapy during their hospital stay compared to less motivated patients (15). A study on depression also reported that patients with relatively high levels of depressive symptoms who were motivated early in their treatment could benefit significantly more from psychosomatic therapy than patients with low levels of motivation (16). Furthermore, psychotherapy, which focuses on motivation, is also widely known (17). All four patients had feelings of insufficiency in their own life situations and thought that the issue lay in others when their pathology was communicated using objective test results. It was suggested that the patients' understanding was deepened by not only words but also numerical values, figures, and explanations using the drawing drawn by the patients themselves. The patients were then able to be introduced to IPT with high motivation from the initial stage of treatment. The supportive and cooperative involvement of many staff members during the examination may have served as the basis for change. This may have resulted in the smooth introduction of the “medical model” used by IPT, which was that “the current condition is due to symptoms, and what is needed is therapy,” increased motivation toward treatment, and higher treatment effects.

Research on employment and interpersonal relationships began in the United States in the mid-1970s, and a recent meta-analysis of studies involving healthcare professionals has shown an association between burnout and working conditions, interpersonal and professional conflicts, mental distress, and poor social support (4). These studies reported correlations between interpersonal relationships and employment in the workplace, but two of the patients in our study did not address interpersonal relationships in the workplace during the sessions. This was probably because the improvements in attachment problems and communication skills brought by IPT resulted in coping skills when associating with people other than significant others, which were learned through treatment. All four patients underwent therapeutic interventions for relationships with their significant others in the process of changing their social roles (gained necessary support in the process of coping with interpersonal conflicts in the family and workplace, and stabilized employment). As noted above, IPT is a treatment that deals with social support itself, so improving symptoms with IPT and restoring social function may be of constant relevance. Reports on IPT that compared treatment as usual (TAU) and group sessions of workplace-related interpersonal psychotherapy (W-IPT) among 27 depression patients have shown that it has significant effects on depressive symptoms as well as in subjective work performance, self-efficacy to return to work, and insomnia (18). A randomized trial that compared eight sessions of W-IPT and TAU in 28 patients with depression exhibited significant improvements and the effectiveness of W-IPT in reducing depressive symptoms, work-ability (WAI), return-to-work attitude (RTW-SE), and the effort-reward ratio (ERI) (19). We anticipate further accumulation of evidence relating to employment and interpersonal relationships.

## Conclusion

We reported four cases of TRD in which IPT was conducted, which resulted in patients returning to work and finding stable employment. The results of various tests performed to formulate the treatment plan, the detailed indications for IPT discussed at the conference, and the motivation for IPT through feedback of the results are the features of this study. IPT, which focuses on relationships with significant others, is a treatment method that not only improves symptoms but also intervenes in social functions, and it can be an effective option when considering support to return to work. With medication control and assessment of depressive symptoms over time, more precise study on the correlation between interpersonal relationships, depressive symptoms, and employment is expected in the future.

## Data Availability Statement

The original contributions presented in the study are included in the article/supplementary material, further inquiries can be directed to the corresponding author/s.

## Ethics Statement

The studies involving human participants were reviewed and approved by Faculty of Medicine Research Ethics Committee, Kyorin University. The patients/participants provided their written informed consent to participate in this study.

## Author Contributions

TK, YT, MK, and YO conceived and designed the manuscript. TK enforced the IPT. All patients were involved in the treatment of these cases. TK, YT, MK, YO, and KW drafted the manuscript. All authors contributed to the article and approved the submitted version.

## Conflict of Interest

The authors declare that the research was conducted in the absence of any commercial or financial relationships that could be construed as a potential conflict of interest.

## References

[1] Rush AJ Trivedi MH Wisniewski SR Nierenberg AA Stewart JW Warden D. Acute and longer-term outcomes in depressed outpatients requiring one or several treatment steps: a STAR^*^D report. Am J Psychiat. (2006) 163:1905–17. 10.1176/ajp.2006.163.11.1905 17074942

[2] DiBernardo A Lin X Zhang Q Xiang J Lu L Jamieson C. Humanistic outcomes in treatment resistant depression: a secondary analysis of the STAR^*^D study. BMC Psychiatry. (2018) 18:352. 10.1186/s12888-018-1920-7 30373547 PMC6206859

[3] Mueller TI Kohn R Leventhal N Leon AC Solomon D Coryell W. The course of depression in elderly patients. Am J Geriat Psychiat. (2004) 12:22–9. 10.1097/00019442-200401000-00003 14729555

[4] Dubale BW Friedman LE Chemali Z Denninger JW Mehta DH Alem A. Systematic review of burnout among healthcare providers in sub-Saharan Africa. BMC Public Health. (2019) 19:1247. 10.1186/s12889-019-7566-7 31510975 PMC6737653

[5] Klerman GL Weissman MM Rounsaville BJ Chevron ES. Interpersonal Psychotherapy Of Depression. New York, NY: Basic Books (1984).

[6] Weissman MM Klerman GL Prusoff BA Sholomskas D Padian N. Depressed outpatients. results one year after treatment with drugs and/or interpersonal psychotherapy. Arch Gen Psychiatry. (1981) 38:51–5. 10.1001/archpsyc.1981.01780260053005 7006558

[7] Frank E Kupfer DJ Thase ME Mallinger AG Swartz HA Fagiolini AM. Two-year outcomes for interpersonal and social rhythm therapy in individuals with bipolar I disorder. Arch Gen Psychiatry. (2005) 62:996–1004. 10.1001/archpsyc.62.9.996 16143731

[8] Markowitz JC Petkova E Neria Y Van Meter PE Zhao Y Hembree E. Is exposure necessary? A randomized clinical trial of interpersonal psychotherapy for PTSD. Am J Psychiatry. (2015) 172:430–40. 10.1176/appi.ajp.2014.14070908 25677355 PMC4464805

[9] National Institute for Health and Care Excellence: Clinical Guidelines. Depression in Adults: Recognition and Management. London: National Institute for Health and Care Excellence (UK) (2009).

[10] Souza LH Salum GA Mosqueiro BP Caldieraro MA Guerra TA Fleck MP. Interpersonal psychotherapy as add-on for treatment-resistant depression: a pragmatic randomized controlled trial. J Affect Disord. (2016) 193:373–80. 10.1016/j.jad.2016.01.004 26799332

[11] Ijaz S Davies P Williams CJ Kessler D Lewis G Wiles N. Psychological therapies for treatment-resistant depression in adults. Cochrane Database Syst Rev. (2018) 5:Cd010558. 10.1002/14651858.CD010558.pub2 29761488 PMC6494651

[12] Buck JN. The H-T-P test. J Clin Psychol. (1948) 4:151–9. 10.1002/1097-4679(194804)4:2&lt;151::AID-JCLP2270040203&gt;3.0.CO;2-O 18869052

[13] Takahashi M. Byouga Test Shindanhou: HTP Test: Bunkyo Shoin. Tokyo (1967).

[14] Hori H Katsuki A Atake K Yoshimura R Nakamura J Baune BT. Risk factors for further sick leave among Japanese workers returning to work after an episode of major depressive disorder: a prospective follow-up study over 1 year. BMJ Open. (2019) 9:e029705. 10.1136/bmjopen-2019-029705 31511285 PMC6747669

[15] Nickel C Tritt K Kettler C Lahmann C Loew T Rother W. Motivation for therapy and the results of inpatient treatment of patients with a generalized anxiety disorder: a prospective study. Wien Klin Wochenschr. (2005) 117:359–63. 10.1007/s00508-005-0334-y 15989116

[16] Nickel C Muehlbacher M Kettler C Tritt K Egger C Lahmann C. [Treatment 1motivation and results of inpatient psychotherapy for women with depressive disorders: a prospective study]. Gesundheitswesen. (2006) 68:11–7. 10.1055/s-2005-859011 16463240

[17] Miller WR Rollnick S. Motivational Interviewing, Third Edition: Helping People Change. New York, NY: Guilford Publications (2012).

[18] Niedermoser DW Kalak N Kiyhankhadiv A Brand S Walter C Schweinfurth N. Workplace-related interpersonal group psychotherapy to improve life at work in individuals with major depressive disorders: a randomized interventional pilot study. Front Psychiatry. (2020) 11:168. 10.3389/fpsyt.2020.00168 32256402 PMC7090238

[19] Schramm E Mack S Thiel N Jenkner C Elsaesser M Fangmeier T. Interpersonal Psychotherapy vs. treatment as usual for major depression related to work stress: a pilot randomized controlled study. Front Psychiatry. (2020) 11:193. 10.3389/fpsyt.2020.00193 32256410 PMC7093578

